# Numerical Simulation and Experimental Validation of Liquid Metal Droplet Formation in a Co-Flowing Capillary Microfluidic Device

**DOI:** 10.3390/mi11020169

**Published:** 2020-02-05

**Authors:** Qingming Hu, Tianyi Jiang, Hongyuan Jiang

**Affiliations:** 1 School of Mechatronics Engineering, Harbin Institute of Technology, West Da-zhi Street 92, Harbin 150001, China; jty_hit@sina.com; 2 School of Mechatronics Engineering, Qiqihar University, Wenhua Street 42, Qiqihar 161006, China

**Keywords:** droplet formation, phase field model, interfacial tension, glass capillary microfluidic device

## Abstract

A two-phase flow axisymmetric numerical model was proposed to understand liquid metal droplet formation in a co-flowing capillary microfluidics device based on a phase field model. The droplet detachment processes were observed in the experiment and are in good agreement with the simulation method. The effects of the viscosities and flowrates of the continuous phase fluid, interfacial tension as well as the wetting property of the metallic needle against the bulk liquid metal on the droplet formation and production rate were numerically investigated. It was found that the droplet diameter decreased with the increment of the viscosities and flowrates of the outer phase carrier fluid. The dispersed phase fluid with high interfacial tension tended to prolong the time for equilibrium between the viscous drag force and interfacial tension on the liquid–liquid fluid surface, delaying the droplet to be pinched off from the capillary orifice and causing large droplet diameter. Finally, the wetting performance of the metallic needle against the liquid metal was explored. The result indicate that the droplet diameter became less dependent on the contact angle while the size distribution of the liquid metal droplet was affected by their wetting performance. A more hydrophilic wetting performance were expected to prepare liquid metal droplet with more monodispersity. The numerical model and simulation results provide the feasibility of predicting the droplet formation with a high surface tension in a glass capillary microfluidic device.

## 1. Introduction

With so many extraordinary physical and chemical properties, such as low melting point, low vapor pressure, high electrical/thermal conductivity and high surface tension, the liquid metals are very useful and highly potential in soft and stretchable electronics [1], heat transfer management devices [2] and functional composites [3], e.g., ultra-soft and compliant electrodes [4], interconnects [5], electrochemical sensors [6], three-dimensional printing [7,8], smart actuators and shape-memory alloys [9]. In particular, liquid metal microdroplets with a symmetrical spatial structure are useful in developing novel microfluidics engineering devices, advanced functional electronics. In addition, the liquid metal microdroplets with highly monodispersity possess tremendous application potentials in various fields of microfluics actuators, periodic structures and optics devices, for instance, microswitches [10,11,12], micropumps [13], micromixer [14], self-powered acceleration sensor [15], radio frequency resonators [16] and reconfigurable optical diffraction gratings [17,18,19]. Therefore, the fabrication of liquid metal droplets with uniform size is of great significance.

It is well known that the droplet size distribution mainly determined by the particular mechanism of droplet generation. Due to the large surface tension and high density, it is difficult to split the bulk liquid metal into microdroplets with the traditionally bulk emulsification method. Several approaches have been developed to fabricate liquid metal microspheres, such as molding [20], sonication [21] and acoustic waves [22]. The molding technique is based on the topographical mold featured by patterned cylindrical reservoirs with which the bulk liquid metal was spreading onto elastomeric sheet and flowed into the reservoirs. With the oxide skin over the droplet surface stabilize the shape firstly, the acid was employed to remove the oxide skin, enabling the totally spherical shape. Despite the simplicity of preparing the liquid metal droplets with the molding, the resulting liquid metal microdroplets size is limited to 100 μm and the molding process is not dynamical adjustable. Simultaneously, it could be time-consuming for large batch production. To improve massive production rates, the sonication technology was adopted to split bulk liquid metal into micro- to nanoscale spheres in the presence of ligands by inserting ultrasonication probe into a nonsolvent. While compared with the molding approach, the sonication method provided limited control over the droplet monodispersity and usually produce liquid metal droplets with a broad droplet size distribution, ranging from less than a few hundred nanometers to several micrometers. Additionally, the bulky sonication device makes it intractable to integrate with other detection and sensing equipment for lab on a chip application. As an alternative strategy, the acoustic wave-induced forces was explored to generate liquid metal microspheres with controllable size by adjusting the interfacial tension of the metal through the external excitation voltage in the acoustic field. The piezoelectric transducer and the signal generator devices maybe increase the cost and the system complexity. Therefore, it is extremely urgent to seek out a preferred technique to produce uniform liquid metal microspheres on demand. Microfluidic technology provides fast, high-throughput and better control over the droplet size distribution, which are useful and highly potential in various applications in many fields such as biochemical and materials science [23,24,25]. Typically, flow-focusing and co-flowing droplet formation mechanism-based droplet microfluidics have been developed for the preparation of uniform-sized liquid metal microspheres [26,27,28,29,30,31,32,33]. When the two neighboring immiscible fluids coming across a small orifice in the microchannel, the flow-focusing based microfluidic chip split the bulk liquid metal into microspheres with the continuous phase fluid pinching off the dispersed phase liquid metal. The droplet diameter and the generation frequency are determined by the balance between the interfacial tension and the viscous shear force exerted by the continuous phase on the dispersed phase [30]. The microsphere size can be tuned by adjusting the shearing rate of two immiscible fluids, which is determined by the flowrate, viscosity of the continuous phase and the chip geometry. The commonly used chip geometry for liquid metal preparation is X-mixer. Michael D. Dickey et al. experimentally studied the formation of galinstan liquid metal microdroplets with high viscosity continuous phase fluid pinching off the bulk liquid metal. They also investigated the influence of flowrates ratios, outer continuous phase fluid viscosities, chip geometries, and interfacial tension on the droplet size [30]. On the other hand, the electric field was implemented to rapidly vary and decrease the liquid metal microdroplets size under the same experimental conditions by changing the interfacial tension of the metal through both electrochemistry and electrocapillarity.

Although flow-focusing-based droplet technology can produce high interfacial tension liquid metal microdroplets with a relative high monodispersity, the fabrication procedures of commonly used polydimethylsiloxane (PDMS) microchannel [34] are complicated and the continuous phase organic solvents may sometimes inflate the soft PDMS microchannel, which may influence the droplet size distribution when the flowrates of the injecting flows are relatively high. As the capillary-based microfluidic device has a strong resistance to organic solvents and aggressive chemical reactions, the droplets can be prepared with less preparation costs and high accuracy by using co-flow devices, which has drawn much attention by research communities. Meanwhile, the strategy of placing wire inside the inlet microchannel was introduced to improve the droplet uniformity and provide better control over the droplet size [35,36,37]. To further improve the monodispersity of generated liquid metal microspheres, we therefore previously put forward a micro-needle induced strategy for the fabrication of liquid metal droplets in a co-flowing capillary microfluidic device in which a stainless steel micro-needle was inserted into the inner liquid metal phase in the glass capillary [31]. The experimental investigation showed that highly uniform liquid metal microdroplets can be obtained.

As the interfacial hydrodynamic behavior of droplet generation is affected by many parameters, mainly including the flowrates of the continuous and dispersed phase, fluid viscosities, interfacial tension, we can dynamically control the dripping mode generation and the corresponding droplet size by adjusting the aforementioned parameters. Usually, the theoretical analysis and experimental investigation was adopted to acquire the flow phenomenon in the capillary-based microfluidic device and predict the droplets sizes. Due to the complexity of theoretical analysis and the high cost of conducting experimental study, the numerical simulation has been performed to investigate such complex phenomenon in the co-flowing device. For instance, Shaowei Li et al. [38] employed a modified level set method to investigate the flow pattern transition and droplet breakup dynamics in a coaxial microchannel. Deng [39] exploited the volume-of-fluid/continuum-surface-force method to simulate the hydrodynamics of oil-in-water droplet formation in a co-flowing capillary device and systematically discussed the effect of interfacial tension, wetting properties of the capillary, and the velocities and viscosities of the two inlet fluids on the droplet size.

However, the current simulation studies mainly concentrated on the water-in-oil or oil-in-water droplet formation, and the majority of studies have focused on the experimental investigation of liquid metal microdroplets formation. The research on the numerical investigation on the liquid metal microsphere formation with microfluidic technology is very sparse, especially with a microfiber inside the inner capillary to induce the stable droplet formation, while it is also of great importance in predicting the droplet size and saving the fabrication cost. Herein, we presented the numerical investigation on the liquid metal microdroplets generation in co-flowing capillary device with a micro-needle in the inlet capillary. The effects of the interfacial tension, wetting properties of the micro-needle, and the viscosities and flowrate of the continuous phases on the droplet size were systematically studied.

## 2. Experiments

### 2.1. Materials

The glycerol (purchased from Aladdin, Shanghai, China) aqueous solution and Galinstan liquid metal (purchased from Sigma, St. Louis, MI, USA) were adopted as the continuous and dispersed phase, respectively. To avoid the spontaneous coalescence of generated liquid metal droplets, the surfactant Poly(vinyl alcohol) (PVA, 87-89 hydrolyzed, average molecular weight (MW), MW = 13,000–23,000) aqueous solution was added into the glycerol solution. The weight ratio of glycerol and 5% PVA aqueous solution was 10:2.9. Therefore, the dynamic viscosity for the two-phase fluids were defined as 0.044 Pa·s and 0.002 Pa·s at room temperature [31], respectively. The interfacial tension between the liquid metal and the glycerol aqueous solution was 0.534 N/m [28]. With a strong chemical attack resistance, a good rigidity and high tensile strength, the 1Cr18Ni19Ti stainless steel micro-needle (Zongsheng, Harbin, China) with a diameter of 70 μm was utilized as the guiding wire.

### 2.2. Experimental Setup

**Figure 1:**
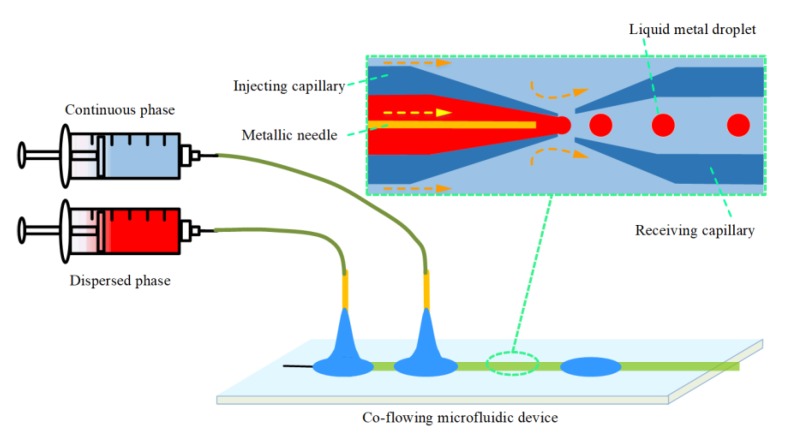
Schematic of the micro-needle induced co-flowing microfluidic experimental setup.

As depicted in Figure 1 (the experimental setup could be found in Figure S1), the capillary-based co-flowing microfluidic device was manufactured by assembling two tapered glass capillaries insides a square channel (AIT Glass, Inc., Largo, FL, USA, 810-9917). The two inner capillaries were concentric aligned with axial spacing of 100 μm, and the metallic needle was inserted into the inlet capillary along the axis of the orifice, with a transverse distance between the micro-needle tip and the center of the capillary orifice of 175 μm. The detailed dimensions and fabrication processes of the micro-needle induced co-flowing glass capillary microfluidic device could be obtained in our previous published paper [31]. The glycerol and liquid metal were supplies separately by two microsyringe pumps (Harvard Apparatus, Holliston, MA, USA) equipped with gastight precision glass syringes (Hamilton, Bonaduz, Switzerland). An optical microscope (CKX41, Olympus, Tokyo, Japan) with a high-speed charge coupled device (CCD) video camera (DP27, Olympus, Japan) connected was utilized to record the flow pattern and the droplet formation in the microdevice.

## 3. Numerical Simulation

### 3.1. Governing Equations

A two-dimensional axisymmetric numerical model was developed to simulate the liquid droplet emulsion formation. The transient flows of the continuous and dispersed phases in the co-flowing capillary are governed by the incompressible Navier–Stokes equation and continuity equation [40].

(1) $$\rho \frac{\partial u}{\partial t}+\rho \left(u\cdot \nabla u\right)=\nabla \cdot \left[-pI+\mu \nabla \left(\nabla u+\nabla u^{T}\right)\right]+F$$

(2) ∇u=0

where *ρ* and **u** are the density and velocity vector, respectively, while *t*, *p* and I are the time, pressure and identity matrix. *μ* denotes the dynamics viscosity of the fluid. *F* signifies body force/source force, mainly includes gravitational force, inertial force and interfacial force. While the droplet diameter is in the order of micro-scale in the co-flowing capillary, the gravitational force and the inertial force can be neglected compared with the interfacial force, The interfacial tension force acting on the liquid–liquid interface between two immiscible fluids can be expressed as follow [41,42]:

(3) F=σκδn

where *σ* is the surface tension coefficient, *k* and *δ* denote the curvature of the interface and the function concentrated across the neighboring immiscible fluids interface, respectively. n is the unit interface normal vector pointing into the droplet, which can be obtained through the flowing equation [42]:

(4) $$n\;=\;\frac{\nabla \phi }{\left|\nabla \phi \right|}$$

where ϕ is the phase field function, which is applied to describe the fluid–fluid interface between two neighboring immiscible mediums and can be calculated by the following advection equations [43].

(5) $$\frac{\partial \phi }{\partial t}+u\cdot \nabla \phi =\nabla \cdot \frac{\gamma \lambda }{\varepsilon^{2}}\nabla \psi$$

(6) $$\psi =-\nabla \cdot \varepsilon^{2}\nabla \phi +\left(\phi^{2}-1\right)\phi$$

where *γ*, *λ* and *ε* are the numerical stabilization parameters, which are adopted to control the interface thickness, mobility tuning and define reinitialization parameter, respectively. To minimize the numerical oscillations, the phase field function ϕ is utilized to smooth the fluid properties across the two neighboring immiscible fluids and track the profile of the liquid–liquid interface [44].

(7) $$\rho =\rho_{\mathrm{continuous}}+\left(\rho_{\mathrm{dispersed}}-\rho_{\mathrm{continuous}}\right)\phi$$

(8) $$\mu =\mu_{\mathrm{continuous}}+\left(\mu_{\mathrm{dispersed}}-\mu_{\mathrm{continuous}}\right)\phi$$

The density *ρ* and dynamics viscosity *μ* in Equation (1) can be obtained with the above Equations (7) the (8). The phase field function denotes fractional volume of the continuous and dispersed phases in a computational cell. Herein, ϕ = 1 and ϕ = 0 represent the cell is filled with dispersed phase and continuous phase, respectively, while 0 < ϕ < 1 indicates the fluid–fluid interface existing in the cell.

### 3.2. Numerical Method

**Figure 2:**
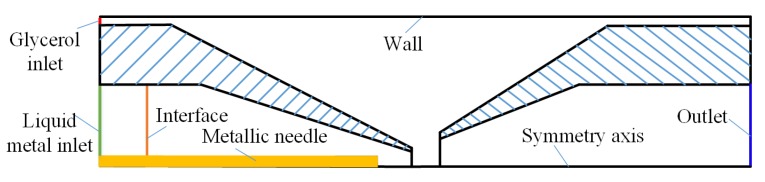
Geometry and boundary condition of liquid metal microsphere formation simulation with the micro-needle induced strategy in co-flowing microchannel.

We conducted the numerical simulation in order to explore the flow pattern and the liquid metal microsphere formation simulation using a commercial software package (COMSOL Inc., Stockholm, Sweden). As depicted in Figure 2, we created an axisymmetric simulation geometry of the flow domain for the three-dimensional co-flowing capillary. The glycerol aqueous solution was introduced into the annular clearance between the outer square capillary and inner circular capillary, while the Galinstan bulk liquid metal was injected into the inner round capillary.

**Figure 3:**
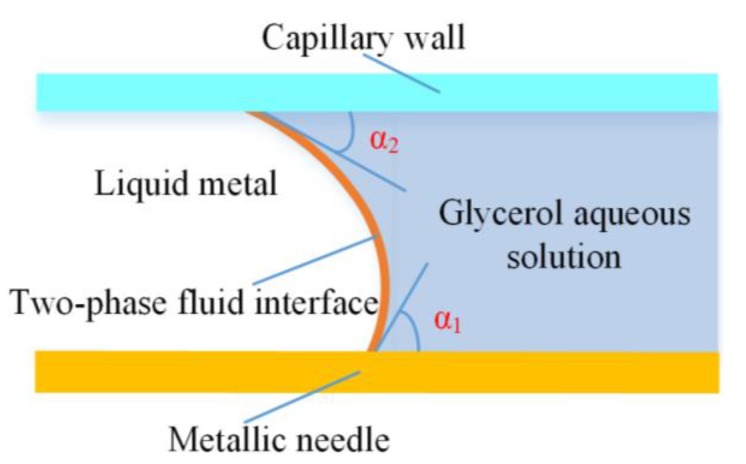
The contact angle between the liquid metal and the metallic needle, and capillary wall, respectively.

The influence of wetted wall on the droplet formation can defined by contact angle between the two neighboring fluids in the simulation. Arising from the effect of metallic affinity, we determined the wetting property of inner phase bulk Galinstan liquid metal on the micro-needle by defining the static contact angle between the liquid metal and the micro-needle, which is shown in Figure 3 as α_1_. On the other side, the contact angle between the liquid metal and the capillary wall was described as α_2_. The initial contact angle between the continuous phase and the micro-needle and the glass wall were determined as pi/4 and 5 pi/18, respectively [45].

Due to the small Reynolds number in the co-flowing capillary microchannel, the laminar flow model was utilized. The phase field function was set as an additional variable in the simulation and adopted to track the two-phase immiscible fluids interface structure. The advection term was used to calculate the momentum and the phase field function. The mobility tunning *λ* parameter was set as 1. The inlets of continuous phase and dispersed phase were defined by the normal inflow speed calculated from volume flowrate, while the outlet was exerted as the opening boundaries with one atmospheric pressure. The capillary microchannel sidewalls and the metallic needle surface were imposed as the non-slip condition. Since the grid may significantly influence the droplet formation simulation result, a local refinement grid was adopted around the contact surfaces between the continuous phase and the inner capillary wall and the micro-needle surface to capture the free surface–surface change more accurately and smoothly. The elements number was 58,044 in the computational domain. In the following calculation, we found that the results variation caused with the mesh refinement is no more than 4%.

## 4. Results and Discussion

### 4.1. Model Validation of Simulation Results

**Figure 4:**
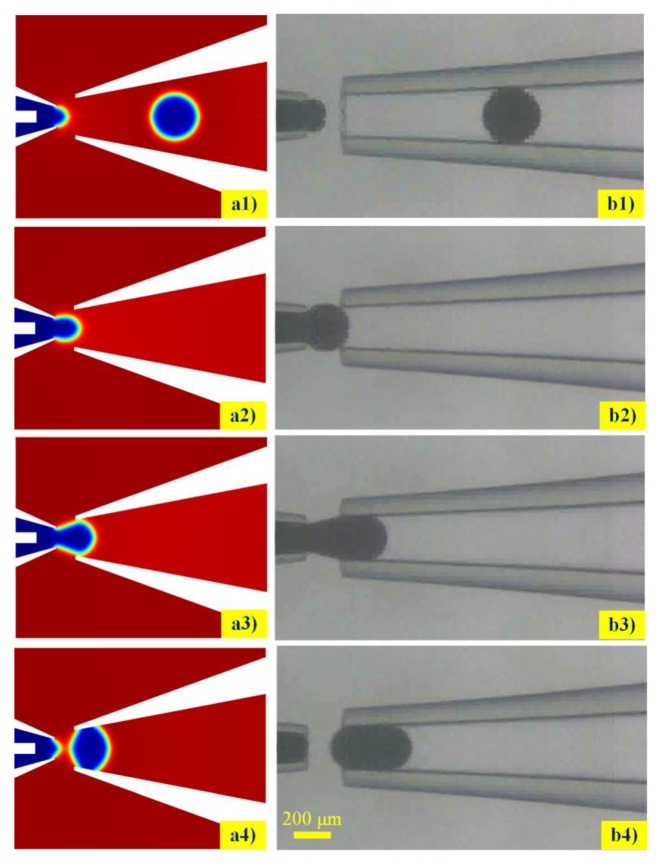
Comparison of liquid metal microdroplets detachment form the capillary tip between simulated (**a1**–**a4**) and experimental (**b1**–**b4**) observation during the droplet formations.

A series of numerical simulations and experiments were conducted to observe the droplet morphology variations during the liquid metal droplet formation. We defined the initial interfacial tension *σ* = 0.534 N/m, the viscosity of the continuous phase and the disperse phase were 0.044 Pa·s and 0.002 Pa·s, respectively. Figure 4 presents the simulated and experimental snapshot of droplet formation process in the coaxial microchannel. On the whole, the simulated two-phase fluids interface movement was in accordance with the experiment observation results, which validated that the phase-field method was capable of predicting the morphological variations during the liquid metal microdroplet formation. Due to the large interfacial tension and high viscosity of the Galinstan liquid metal, the droplet initially attached to the capillary tip and kept growing with the bulk liquid metal continuously being injected, which, in some cases, accentuated the effect of contact line dynamics. The necking of the bulk liquid metal was initiated once the glycerol aqueous solutions was intruded near the inlet capillary orifice. Simultaneously, the neck became thin with the increment of the viscous drag force. The liquid metal droplet detached from the capillary orifice when the viscous drag force was large enough to oppose the action of the surface tension. As the Galinstan liquid metal had a large surface tension, the produced droplet size was relatively bigger than the traditional oil in water or water in oil droplet module under the same circumstances [28].

**Figure 5:**
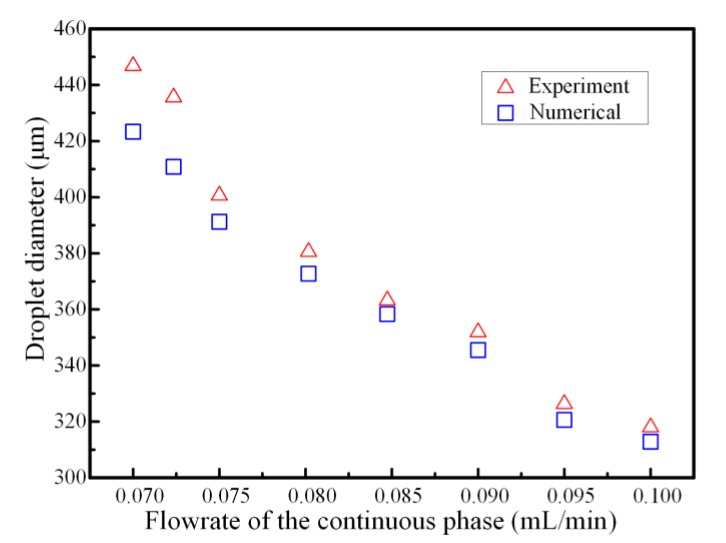
Comparison of the droplet diameter between numerical simulations and experimental results for different flowrates of continuous phase when the flowrate of dispersed phase was fixed as 10 μL/min.

To further validate the effectiveness of the proposed phase-field method-based two-phase flow axisymmetric numerical model, droplet formation simulations were conducted for different flowrates of continuous phase when the flowrate of dispersed phase was defined as 10 μL/min, ranging from 0.070 to 0.100 mL/min. The droplet diameters in the experiment were compared with the numerical results. It was predicted from Figure 5 that droplet size decreased as the flowrate of the continuous phase increased. The variation tendency between the droplet size and the flowrate of the continuous phase was also in agreement with the experimental results published by Hutter [29]. The droplet diameter generated with the simulation was 312.3 μm when the continuous phase flowrate and the dispersed phase flowrate were 100 μL/min and 10 μL/min, respectively, which was almost consistent with the experimental result of 318 μm under the same circumstances. Additionally, the droplet sizes obtained from the experiment were slightly larger than the simulation results, whereas the discrepancy decreased with the increasing flowrate of the glycerol aqueous solution. This could be attributed to the velocity variation fluctuation of the injecting bulk liquid metal stream during the experiment. Both simulation and experiment indicated that the generated liquid metal microspheres were highly monodispersed.

### 4.2. Effect of Interfacial Tension

**Figure 6:**
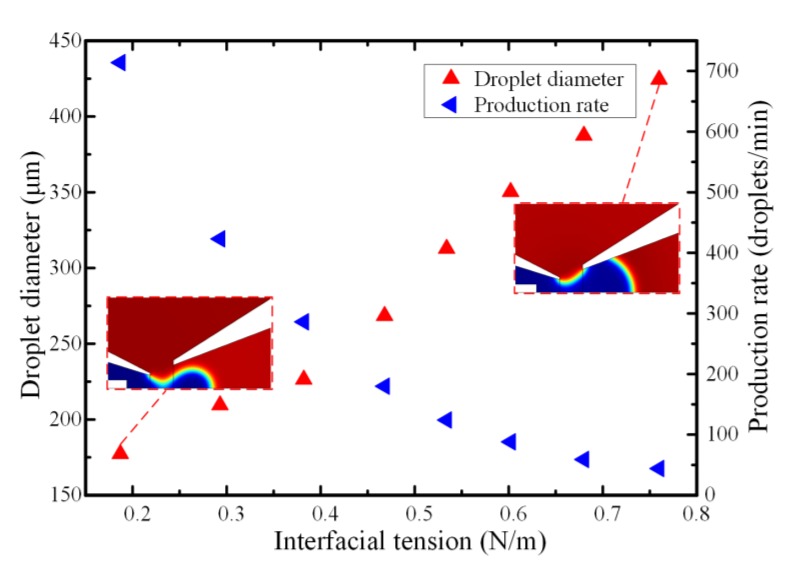
The effect of interfacial tension on the droplet diameter and generation frequency when the flowrate of continuous and dispersed phases were 100 μL/min and 10 μL/min, respectively.

The interfacial tension, which was the dominating force at the microscale, was of vital role in the process of emulsification. When the two immiscible fluids intimately contacted with each other, the liquids tended to be spherical to maintain the smallest energy on the two-phase fluid interface [46]. The breakup mechanism and stability of the droplets were affected by two neighboring fluids interfacial tension in co-flowing capillary microfluidic device. Especially for the liquid metal with high surface tension, the interfacial tension could keep the liquid metal microsphere attached at the capillary tip to avoid being detached from the incoming dispersed phase stream. Figure 6 depicts the relationship between the interfacial tension on the microdroplet formation for the fixed flowrate of continuous phase and dispersed phases being as 100 μL/min and 10 μL/min, respectively. Compared with the traditional oil phase droplet formation, the simulation resulted show that the liquid metal droplet diameter was much larger and the production rate was lower increased with the increment of interfacial tension under the same circumstance. As the interfacial tension could always keep the droplet attaching to the capillary tip, the droplet volume became larger and larger with the incoming continuous phase bulk liquid metal. Then, the droplet was detached from the orifice when the equilibrium between the interfacial tension and the viscous drag force was broken. On the other hand, the size of produced microdroplets varied inversely with the dimensionless capillary number, *Ca* = *Vμ*/*γ* (where *γ* was the surface tension at the interface between the bulk liquid metal and the carrier fluid, *V* and *μ* were the characteristic velocity and dynamic viscosity of the glycerol aqueous solution, respectively). Namely, changing the interfacial tension at the two-phase surface could allow us to adjust the liquid metal microspheres diameter. Therefore, it could be accountable that the time for reaching the equilibrium state was much longer with the increasing liquid metal surface tension, resulting in a longer droplet length and a larger diameter.

### 4.3. Effect of Viscosity of Continuous Phase Fluid

**Figure 7:**
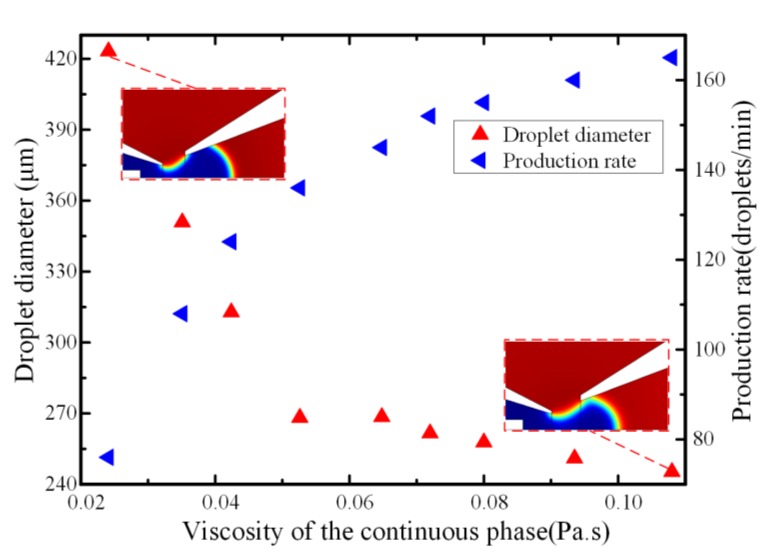
The effect of the viscosity of continuous phase on the droplet diameter and generation frequency when the flowrate of continuous and dispersed phases were 100 μL/min and 10 μL/min, respectively.

The incoming fluid viscosity could affect the flow pattern and dynamic behavior of droplet formation. When the viscosity discrepancy exceeded a certain threshold, the equilibrium state between the interfacial tension and the drag force was broken. The droplet could be prepared smoothly when there were matched viscosities between the continuous phase and the disperse phase [47]. The viscosity effect on droplet formation in a micro-needle induced co-flowing microfluidic device was investigated numerically. At a lower continuous fluid viscosity, which was about 10 times the inner phase, the fluid pair behaved more like a single fluid and the shearing force acting on the bulk liquid metal were much lower, causing a long time for the droplets to be pinched off from the capillary tip and hence produced a larger droplet diameter. By increasing the viscosity of continuous fluid, the viscous shear stress acting on the boundary of neighboring immiscible fluids increased, causing droplets formation to occur much quickly. The greater discrepancy between the neighboring fluids viscosities, the bigger viscous shear acting on the fluid interface, resulting in a shorter detachment time and much smaller droplets. The results (shown in Figure 7) indicate that the liquid metal droplet diameter was decreased from 423.2 to 245.3 μm with the increase of viscosity ratios from 10 to 55 under the same flow condition *Q_w_* = 100 μL/min, *Q_o_* = 10 μL/min. The effect of increasing the continuous phase viscosity also increased the rate of droplet production. With the decreasing in liquid metal droplet volumes as the outer phase fluid viscosity increased, the droplet generation frequency increased to account for the decreased droplet volume. Additionally, the bulk liquid metal was sheared off more quickly due to the larger continuous fluid viscosity, leading to an increase in the droplet frequency.

### 4.4. Effect of the Flowrates of Continuous Phase

**Figure 8:**
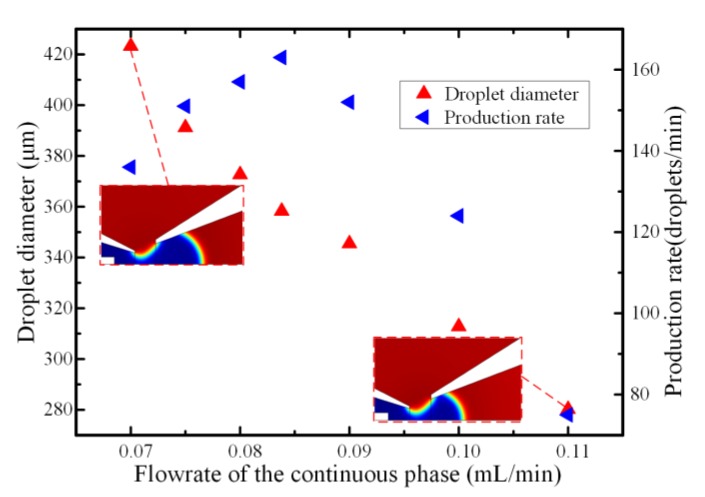
The effect of the flowrate of the continuous phase on the droplet diameter and generation frequency when the flowrate of dispersed phase and the viscosity of continuous phase were set as 10 μL/min and 0.044 Pa·s, respectively.

The droplet size could be controlled by adjusting the flowrate of the inlet immiscible flows. The lower continuous fluid flowrate led to a larger droplet size and a lower generation frequency. Figure 8 depicts the effect of continuous phase flowrates on the droplet formation with the dispersed phase flowrate fixed at 10 μL/min and the viscosity of continuous phase were set as 10 μL/min and 0.044 Pa·s, respectively. The droplet diameter decreased with the increase of the continuous phase flowrate, which was in good accordance with the relationship between the capillary number and the droplet size. Simultaneously, it could be observed from the simulation snapshot that the droplet length became shorter as the continuous phase flowrate increased. This could be ascribed to the higher velocity gradient generated on the liquid metal–glycerol interface under the higher continuous fluid velocity, which caused a higher viscous drag force acting on the droplet and hence, smaller droplets could be obtained. When the flowrate of the glycerol aqueous solution was less than 0.085 mL/min at the fixed dispersed phase flowrate of 10 μL/min, the droplet production augmented significantly linearly proportionally to the flowrate. That was because production frequency and the droplet volume must be inversely related to maintain the flow continuity in the capillary microchannel under the low outer phase fluid flowrate, which was also in good accordance with the previously published conclusion [48]. However, at the fixed dispersed phase flowrate, the bulk liquid metal stream was suppressed and could not smoothly flow out of the inner capillary when the flowrate of the continuous phase was large. The competition between the drag force and the surface tension on the liquid metal–glycerol aqueous solution interface was broken with the flowrate of the continuous phase above a certain critical value. The liquid metal microsphere was detached from the capillary tip and then the liquid metal stream was retracted back into the inner capillary due to the high continuous fluid velocity and flow instability. Therefore, the hydrodynamic instability of the liquid metal stream is significant during the necking and pinch-off stage when the flowrate of the continuous phase is high, leading to longer droplet formation time interval and hence, a lower droplets generation frequency. The simulation results of the relation between the droplet production rates and the flowrate are also in agreement with the experimental results of the previous findings [29].

### 4.5. Effect of the Wetting Property of the Micro-Needle

**Figure 9:**
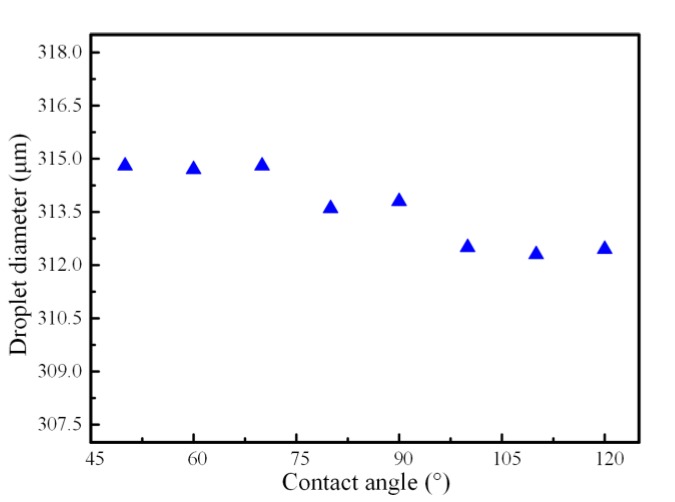
The effect of contact angle on the droplet diameter when the flowrates of the continuous phase and the dispersed phase were fixed at 100 μL/min and 10 μL/min, respectively.

As the micro-needle was inserted into the inner capillary, the wetting properties might influence the droplet diameter and the size distribution. We previously defined the contact angle among the metallic needle, bulk liquid metal and the glycerol solution, and herein, numerically investigated the droplet diameter with respect to the contact angle when the viscosity of continuous phase was 0.044 Pa·s. Figure 9 depicts the effect of the contact angle α_1_ on the droplet diameter with a fixed contact angle α_2_. On the whole, the droplet diameter declined from 314.8 μm to 312.2 μm with the contact angle increased from 50° to 120° when the flowrates of the continuous phase and the dispersed phase were fixed at 100 μL/min and 10 μL/min, respectively. Namely, the contact angle had a slight influence on the droplet size. However, after the statistical analysis of the simulated generated droplet, the variance of the droplet diameter normal distribution for the contact angle of 120° was 2.26, which is much smaller than 8.35 with a contact angle of 50°. That means the produced droplet at larger contact angle has better monodispersity compared with the smaller one. It could be worth noting that the micro-needle inside the capillary had a better wettability performance with a larger contact angle, which is good for droplet movement and adsorption on the microfiber [37,49,50]. Therefore, the flow stability of droplet formation was affected at a lower contact angle and a droplet diameter with a better distribution could be obtained with a relatively large contact angle.

## 5. Conclusions

In summary, numerical simulations of the liquid metal microspheres formation in a micro-needle induced co-flowing capillary microfluidic device were conducted using the phase-field method. The reasonableness of the computation model was experimentally validated. The relationship between the droplet size and generation frequency with respect to some important factors were numerically investigated, mainly including the interfacial tension, fluid viscosities, wetting property of the micro-needle against the bulk liquid metal and flowrates. The results demonstrate that the breakup mechanism of the liquid metal with a high surface tension was strongly dependent on the competition between the viscous drag force induced by fluid flow of the continuous phase and the interfacial tension on the two-phase fluid interface. On the whole, with the increasing viscosity and flowrate of the continuous carrier flow, a higher viscous drag force was generated and hence, the detachment time for the droplet formation was diminished, leading to the fabrication of smaller liquid metal droplets with a high generation frequency. The smaller capillary number determined by the higher interfacial tension delayed the balance between the drag force and the interfacial tension for the liquid metal droplet to be pinched off from the capillary tip. Accordingly, larger liquid metal droplets were prepared with more inner phase fluid flowing into the droplet under high surface tension. Although the contact angle of micro-needle against liquid metal had a slight influence on the droplet diameter, the size distribution was affected by the contact angle. A droplet of a more uniform size could be obtained when the contact angle was larger. That was probably attributed to the better wettability between the round metallic needle and the bulk liquid metal. The presented axisymmetric numerical model and the simulation results above could provide a reliable prediction for preparation of droplet in a controllable way in co-flowing microfluidic devices.

## Supplementary Materials

The following are available online at https://www.mdpi.com/2072-666X/11/2/169/s1, Figure S1: The image of the experimental setup.
