# Supplementary material for: Numerical Simulation and Experimental Validation of Liquid Metal Droplet Formation in a Co-Flowing Capillary Microfluidic Device
Source: Micromachines (Basel). 2020 Feb 5;11(2):169. doi: 10.3390/mi11020169 (PMC7074579; doi:10.3390/mi11020169)
Supplement: Supplementary file 1 [file micromachines-11-00169-s001.pdf]

micromachines

Supplementary Materials

Numerical Simulation and Experimental Validation of Liquid Metal Droplet Formation in a Co-Flowing Capillary Microfluidic Device

Qingming Hu, Tianyi Jiang and Hongyuan Jiang

Section S1. The Image of the Experimental Setup.

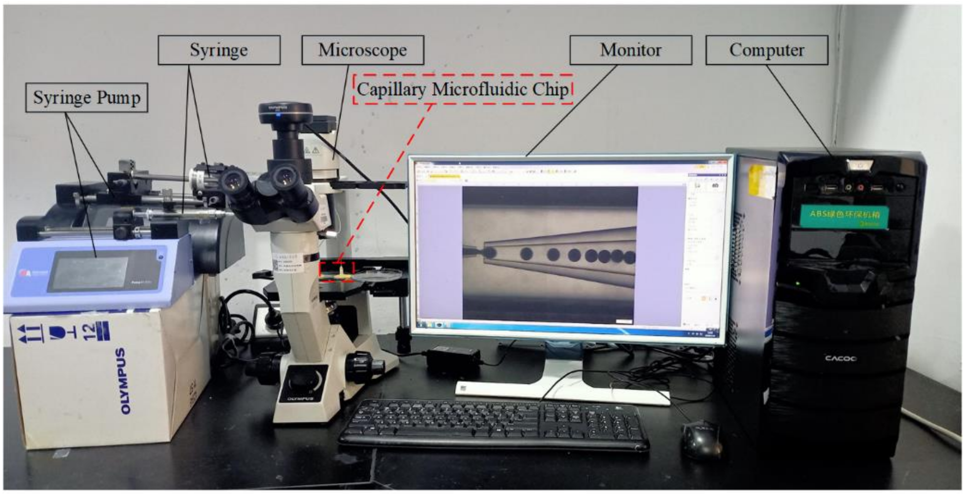

Figure S1. The image of the experimental setup.
